# Long-term safety of long-acting octreotide in patients with diabetic retinopathy: results of pooled data from 2 randomized, double-blind, placebo-controlled phase 3 studies

**DOI:** 10.1007/s12020-017-1448-5

**Published:** 2017-11-07

**Authors:** Rosario Pivonello, Giovanna Muscogiuri, Geoffrey Holder, Michaela Paul, Severine Sarp, Anastasia Lesogor, Pierre Jordaan, Johannes Eisinger, Annamaria Colao

**Affiliations:** 1 Dipartimento di Medicina Clinica e Chirurgia, Sezione di Endocrinologia, Università Federico II di Napoli, Naples, Italy; 2 Novartis AG, Basel, Switzerland

**Keywords:** Diabetic retinopathy, Octreotide, Long-term safety, Renal function, Hepatic function, Cardiac function

## Abstract

**Purpose:**

Octreotide (OCT) has been successfully used for treatment of acromegaly and neuroendocrine tumors for more than 30 years. However, long-term safety of OCT has not been documented in placebo-controlled setting. This present analysis pooled safety data from two similarly-designed, randomized, and placebo-controlled studies to evaluate long-term safety of long-acting OCT (20, 30 mg); targeted post-hoc analyzes focused on cardiac, hepatic, and renal safety.

**Methods:**

Two studies (NCT00131144, NCT001308450) were conducted in patients with diabetic retinopathy (OCT20 = 191, OCT30 = 348, placebo = 347). In this analysis, patients were stratified based on baseline glomerular filtration rate. Hepatic, cardiac, and renal adverse events (AEs) were identified by standardized MedDRA queries.

**Results:**

Median duration of exposure was >3.5 years. Most common AEs reported with OCT were diarrhea, cholelithiasis, hypoglycemia, nasopharyngitis, and hypertension. Incidence of cardiac events (QT prolongation and arrhythmia) with OCT20 and OCT30 were comparable to placebo (OCT20, RR = 1.11 [95% CI, 0.61–2.03]; OCT30, RR = 1.09 [95% CI, 0.70–1.68]). For ECG findings, changes in QTcF were similar in treatment groups, and outliers did not exceed 480 ms. Incidence of cardiac ischemia was lower with OCT than placebo (OCT20 = 12.6%, OCT30 = 10.6%, placebo = 15.3%). Incidence of liver-related AEs was higher with OCT30 than placebo (RR = 2.04 [95% CI, 1.28–3.26]); incidences were comparable with OCT20 and placebo (RR = 1.50 [95% CI, 0.69–3.25]). Overall incidences of renal AEs were comparable between treatment groups (OCT20 = 5.8%; OCT30 = 6.3%; placebo = 7.2%). Drug-related SAEs were reported more frequently with OCT (OCT20 = 7.9%; OCT30 = 10.1%; placebo = 3.5%); predominantly gallbladder-related, GI-related, and hypoglycemia.

**Conclusions:**

The results from these long-term placebo-controlled studies confirm the established safety profile of long-acting OCT, in particular low risk of cardiac, hepatic and renal toxicity in a high-risk population.

## Introduction

Octreotide (SMS 201–995, octreotide acetate, Sandostatin^®^, Novartis) is a somatostatin analogue (SSA) with a therapeutic use in pathophysiological states associated with excessive hormone production and secretion [1–4].

With extensive clinical and real-world use over the past 30 years, octreotide is considered to have a favorable benefit-risk profile in acromegaly and neuroendocrine tumors [5–8]. The majority of adverse events (AEs) are due to the octreotide-induced imbalance of hormonal pathways, most of which occur in the gastrointestinal (GI) tract manifesting commonly in diarrhea, flatulence, abdominal pain and nausea. Other AEs associated with octreotide include hyperglycemia and abnormalities of cardiac rhythm. Hyperglycemia is a common reaction which may be caused by inhibition of insulin secretion. Patients with acromegaly often exhibit underlying insulin resistance because of their excess of growth hormone, and the degree of impairment in glucose homeostasis potentially brought about by SSA treatment is debated in the literature [7, 9, 10, 11]. Abnormalities of cardiac rhythm are clinically relevant in acromegaly. Octreotide has been shown to decrease the heart rate and prolong the QT interval, an important risk factor for ventricular arrhythmias. Although this may be perceived as a treatment risk, there is, however, some evidence that octreotide may have a beneficial cardiac effect in patients with acromegaly with a reduction, and in some cases, normalization of QT intervals [12] and with improvement of cardiac performance and hypertension [13, 14].

Long-term safety of octreotide has been the subject of several published studies and reviews; however, findings associated with octreotide treatment in placebo-controlled studies exceeding 30 months have not been documented. Therefore, the results of two randomized double-blind, placebo-controlled, parallel-group studies present a unique opportunity to assess the safety of octreotide in a vulnerable patient population (diabetic patients with microvascular disease) with a long-term follow-up period over 2 years.

Octreotide is not currently approved for the treatment of diabetes mellitus or diabetic retinopathy; however, patients with diabetes comprise some of the populations with approved conditions. The results from the studies presented here provide critical insight into the safety and tolerability of long-term octreotide use in a population with a high cardiovascular risk.

## Methods

### Description of the pooled trials

Two randomized, double-blind, placebo-controlled studies were conducted between 1999 and 2006 in patients with diabetic retinopathy with the primary objective of determining whether long-acting octreotide, administered at 20 or 30 mg every 4 weeks, delayed the time to progression of diabetic retinopathy according to defined endpoints on the Early Treatment Diabetic Retinopathy Study final retinopathy severity scale (study 1: CSMS995H0802 [NCT00131144] and study 2: CSMS995H0804 [NCT00130845]). This present analysis pooled the safety data of these two studies to evaluate the overall long-term safety and also in the sub-populations defined by the baseline level of renal function. In addition, the targeted post-hoc analyses also focused on cardiac, hepatic, and renal safety.

Patient population and study design were similar for both studies that differed only in the recruitment region and number of investigational treatment arms (study 1: Europe, octreotide 20 mg [OCT20] and octreotide 30 mg [OCT30]; study 2: North America and South America, OCT30). The studies enrolled male and female patients with type 1 diabetes or type 2 diabetes, irrespective of insulin dependence, between 18 and 70 years with an HbA1c < 13.0%. Patients had moderately severe or severe non-proliferative diabetic retinopathy or low risk proliferative diabetic retinopathy. The main exclusion criteria were a history of brittle diabetes or hypoglycemia unawareness, symptomatic gallstones without a cholecystectomy, a positive pregnancy test, or prior treatment with octreotide or another SSA. Overall, 898 patients were randomized, 585 in study 1 (*N* = 195 on OCT20, *N* = 197 on OCT30, and *N* = 193 on placebo) and 313 patients in study 2 (*N* = 155 on OCT30, and *N* = 158 on placebo).

### Compliance with ethical standards

Study 1 and study 2 were approved by the institutional review boards of the respective institutions prior to patient enrollment. All procedures performed in study 1 and study 2 involving human participants were in accordance with the ethical standards of the institutional and/or national research committee and with the 1964 Helsinki declaration and its later amendments. Informed consent was obtained from all individual participants included in the study.

### Safety data

All analyses were based on the safety analyzable population (SAF), defined as patients who received at least one dose of trial medication and for whom one or more post-baseline safety assessments were obtained. The safety population for study 1 consisted of 578 patients (*N* = 191 on OCT20, *N* = 196 on OCT30, and *N* = 191 on placebo) and for study 2 consisted of 308 patients (*N* = 152 on OCT30, and *N* = 156 on placebo). The safety assessments included laboratory data, electrocardiogram (ECG), vital signs, and gallbladder ultrasound. Single 12-lead ECGs were collected at baseline and at the end of treatment. During the study, ECGs were performed if they were clinically indicated by the patient’s symptomatology (as per investigator opinion). All ECGs were assessed centrally by an ECG core laboratory retrospectively for central blinded assessment. For this present analysis, data from the two studies were pooled. Adverse events of special interest (hepatic, cardiac, and renal events) were identified by standardized MedDRA queries (SMQs) based on MedDRA version 18.0, and multiple AEs within the same SMQ were counted only once per patient.

### Sub-populations (based on baseline renal status)

The diabetes population under study was an opportunity to compare and analyze safety data in patients with impaired as well as normal renal function. Accordingly, analyses were presented in the overall SAF population and by sub-populations defined by renal function at baseline, either as normal or mildly impaired renal function (baseline estimated glomerular filtration rate (eGFR) ≥ 60 mL/min/1.73 m^2^), and for impaired renal function (baseline eGFR < 60 mL/min/1.73 m^2^) (Table 1). Renal function was assessed by the abbreviated modification of diet in renal disease eGFR formula [15].

**Table 1:** Baseline eGFR (mL/min/1.73 m^2^) by treatment group and sub-population

Population	Long-acting octreotide		Placebo	Total
	30 (mg)	20 (mg)		
Baseline eGFR ≥ 60 mL/min/1.73 m^2^				
*N*	237	133	238	608
Mean (SD)	74.8 (9.43)	72.8 (9.37)	73.2 (8.52)	73.7 (9.09)
Median	74.3	70.9	72.7	73.1
Min–max	60–104	60–102	60–98	60–104
Baseline eGFR < 60 mL/min/1.73 m^2^				
*N*	110	58	108	276
Mean (SD)	49.2 (8.53)	48.9 (10.16)	49.1 (9.29)	49.1 (9.16)
Median	50.9	52.1	52.4	51.7
Min–max	18–59	21–60	12–60	12–60
All patients				
*N*	347	191	346	884
Mean (SD)	66.7 (15.01)	65.6 (14.62)	65.7 (14.19)	66.0 (14.60)
Median	68.1	65.8	67.2	67.1
Min–max	18–104	21–102	12–98	12–104

### Statistical analysis

Baseline values were defined as the last non-missing value prior to the first dose of study treatment. Adverse event summaries include treatment-emergent AEs, occurring up to and including 90 days after the date of last study medication. For the AEs of special interest, relative risk (RR, risk ratio of incidence on octreotide treatment vs. placebo) estimates and 95% confidence intervals (CIs) were calculated using Mantel-Haenszel method and adjusted for study as stratification factor. Pairwise comparisons of OCT30 vs. placebo and OCT20 vs. placebo for each SMQ were performed overall and in the sub-population based on baseline renal status. For the ECG analysis, the ECG recorded at the end of study visit (EOS) was considered as the last on-treatment ECG. The mean, central tendency change for the ECG parameters from baseline to EOS, as well as the number and percentages of ECG events of interest (categorical changes) were summarized.

## Results

### Demographics and baseline characteristics

All patients had a history of diabetes for ≥10 years (type 2 78.4% overall; type 1 21.6% overall), and current diabetic retinopathy. The majority of patients were overweight (body mass index, median 28.7 kg/m^2^ overall), male (68%), and Caucasian (>80% in all treatment groups and sub-population), and between 51 and 65 years. The demographics and baseline characteristics were mostly similar between the two studies and treatment groups (OCT30, OCT20, and placebo in study 1, and OCT30 and placebo in study 2). Hypertension was the most common comorbidity in all treatment groups. A summary of the baseline characteristics is presented in Table 2.

**Table 2:** Baseline patient characteristics

Patient characteristics	Long-acting octreotide		Placebo	Total
	30 mg *N* = 348	20 mg *N* = 191	*N* = 347	*N* = 886
Age (years), median (min–max)	56.0 (20–77)	56.0 (24–70)	57.0 (23–75)	57.0 (20–77)
Male, *N* (%) Female, *N* (%)	230 (66.1) 118 (33.9)	142 (74.3) 49 (25.7)	228 (65.7) 119 (34.3)	600 (67.7) 286 (32.3)
BMI, median (kg/m^2^) (min–max)	28.7 (18–58)	27.8 (19–50)	29.1 (19–56)	28.7 (18–58)
Summary of diabetes characteristics				
Type 1, *N* (%)	80 (23.0)	35 (18.3)	76 (21.9)	191 (21.6)
Type 2, *N* (%)	268 (77.0)	156 (81.7)	271 (78.1)	695 (78.4)
Duration ≥ 15 years, *N* (%)	155 (44.5)	70 (36.6)	161 (46.4)	386 (43.6)
HbA1c (%), median (min–max)	8.10 (4.8–13.6)	8.00 (4.0–12.5)	8.50 (4.7–15.2)	8.20 (4.0–15.2)
Insulin usage, (yes) *N* (%)	215 (61.8)	124 (64.9)	219 (63.1)	558 (63.0)
Urinary albumin, median (g/L) (min–max)	0.0170 (0.0047–3.5016)	0.0187 (0.0047–2.5230)	0.0353 (0.0047–2.5125)	0.0212 (0.0047–3.5016)
Summary of risk factors				
Current smoker, *N* (%)	54 (15.5)	46 (24.1)	64 (18.4)	164 (18.5)
Systolic blood pressure, median (min–max)	140.0 (80–220)	145.0 (100–200)	140.0 (94–211)	140.0 (80–220)
Diastolic blood pressure, median (min–max)	80.0 (40–110)	80.0 (57–105)	80.0 (50–114)	80.0 (40–114)
Hypertension, (yes) *N* (%)	221 (63.5)	109 (57.1)	209 (60.2)	539 (60.8)
Dyslipidemia, (yes) *N* (%)	10 (2.9)	8 (4.2)	9 (2.6)	27 (3.0)

Percentage are based on *N* = total number of subjects in the treatment group

*BMI* body mass index = weight/(height^2^), *HbA1c* hemoglobin A1c

### Treatment exposure

The median duration of exposure to treatment was comparable between the treatment groups: OCT20 = 188 weeks; OCT30 = 184 weeks; and placebo = 204 weeks. Overall, approximately 40% of the patients in all the three treatment groups had duration of exposure greater than 208 weeks; the maximum duration of exposure to treatment was 300 weeks (~5.7 years).

### Safety evaluation

Overall, the most common AEs reported in the OCT20, OCT30, and placebo groups, respectively were diarrhea (42.9% [82/191], 58.0% [202/348], and 21.0% [73/347]); cholelithiasis (40.8% [78/191], 44.3% [154/348], and 19.6% [68/347]); hypoglycemia (17.8% [34/191], 29.6% [103/348], and 23.6% [82/347]); nasopharyngitis (18.8% [36/191], 24.1% [84/348], and 28.8% [100/347]); and hypertension (23.0% [44/191], 20.7% [72/348], and 23.9% [83/347], Table 3).

**Table 3:** Most frequent adverse events by preferred term, ≥10% in any long-acting octreotide treatment group

Adverse events	Long-acting octreotide		Placebo
	30 mg *N* = 348 *n* (%)	20 mg *N* = 191 *n* (%)	*N* = 347 *n* (%)
Total patients with an AE	341 (98.0)	189 (99.0)	337(97.1)
Diarrhea	202 (58.0)	82 (42.9)	73 (21.0)
Cholelithiasis	154 (44.3)	78 (40.8)	68 (19.6)
Hypoglycemia	103 (29.6)	34 (17.8)	82 (23.6)
Nasopharyngitis	84 (24.1)	36 (18.8)	100 (28.8)
Hypertension	72 (20.7)	44 (23.0)	83 (23.9)
Influenza	52 (14.9)	28 (14.7)	70 (20.2)
Nausea	56 (16.1)	17 (8.9)	53 (15.3)
Macular edema	46 (13.2)	21 (11.0)	43 (12.4)
Abdominal pain	51 (14.7)	16 (8.4)	29 (8.4)
Diabetic nephropathy	27 (7.8)	32 (16.8)	32 (9.2)
Back pain	37 (10.6)	20 (10.5)	39 (11.2)
Vitreous hemorrhage	38 (10.9)	18 (9.4)	62 (17.9)
Pain in extremity	40 (11.5)	16 (8.4)	38 (11.0)
Headache	42 (12.1)	12 (6.3)	43 (12.4)
Vomiting	42 (12.1)	12 (6.3)	40 (11.5)
Cough	41 (11.8)	11 (5.8)	40 (11.5)
Cataract	26 (7.5)	24 (12.6)	36 (10.4)
Dizziness	38 (10.9)	11 (5.8)	35 (10.1)
Anemia	44 (12.6)	5 (2.6)	34 (9.8)
Flatulence	43 (12.4)	6 (3.1)	12 (3.5)
Urinary tract infection	36 (10.3)	12 (6.3)	33 (9.5)

Adverse events led to study drug interruption in 12.0% (23/191) patients on OCT20, 13.8% (48/348) patients on OCT30, and 8.1% (28/347) of patients receiving placebo. Adverse events (suspected to be study drug related) led to discontinuation in 8.9% (17/191) of patients on OCT 20, 16.7% (58/348) of patients on OCT30, and 2.6% (9/347) of patients on placebo. Adverse events (not suspected to be study drug related) led to discontinuation in 10.5% (20/191) of patients on OCT20, 6.9% (24/348) of patients on OCT30, and 8.1% (28/347) of patients on placebo. Neoplasms (benign, malignant and unspecified) were reported in a higher number of patients in the placebo groups in both the studies (study 1: OCT20 = 2.6% [5/191], OCT30 = 1.0% [2/196], placebo = 4.2% [8/191]; study 2: OCT30 = 2.6% [4/152] and placebo = 4.5% [7/156]).

The rate for hyperglycemia was comparable between octreotide treatment groups and placebo (OCT20 = 8.9% [17/191]; OCT30 = 9.8% [34/348]; placebo = 8.6% [30/347]); while the OCT30 group experienced higher rates of hypoglycemia compared with OCT20 and placebo groups (OCT20 = 17.8% [34/191]; OCT30 = 29.6% [103/348]; placebo = 23.6% [82/347]). However, events related to diabetic neuropathy were numerically more frequent in the OCT20 group compared with the OCT30 and placebo groups (OCT20 = 5.2% [10/191]; OCT30 = 2.3% [8/348]; placebo = 2.6% [9/347]); while peripheral neuropathy and paresthesia were more frequent in the placebo group compared with the octreotide groups (paresthesia: OCT20 = 2.1% [4/191]; OCT30 = 1.1% [4/348]; placebo = 2.9% [10/347]; neuropathy peripheral: OCT20 = 0.5% [1/191]; OCT30 = 2.3% [8/348]; placebo = 4.6% [16/347]). Hypercholesterolemia was less prevalent with octreotide than with placebo (OCT20 = 2.6% [5/191]; OCT30 = 5.2% [18/348]; placebo = 13.3% [46/347]). The mean standard deviation [SD] change in weight (kg) from baseline to last value on study was higher in the placebo group vs. the octreotide treatment groups (OCT20 = −0.66 [7.179]; OCT30 = 0.24 [7.151]; placebo = 2.43 [6.773]).

### Safety topics of special interest

**Fig. 1:**
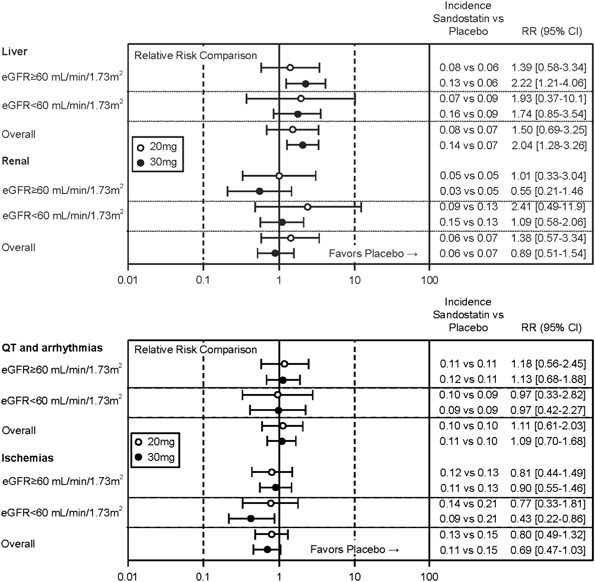
Forest plot of overall relative risk of incidence of hepatic, renal, and cardiac adverse events (sub-populations defined by renal function and all patients). Incidence calculated as number of subjects with any treatment-emergent AE divided by number of subjects at risk. Mantel-Haenszel estimate adjusted for study as stratification factor is used for RR and 95% confidence interval

The incidence of cardiac, renal, and hepatic AEs, by SMQ, is summarized in Table 4, and RR estimates are presented as forest plots in Fig. 1.

**Table 4:** Hepatic, cardiac, and renal adverse events by treatment group and sub-populations defined by baseline eGFR

Adverse events	Long-acting octreotide		Placebo
	30 mg *n* (%)	20 mg *n* (%)	*n* (%)
Baseline eGFR ≥ 60 mL/min/1.73 m^2^			
*N*	237	133	238
Cardiac, QT, and arrhythmias	28 (11.8)	14 (10.5)	25 (10.5)
Cardiac, ischemias	27 (11.4)	16 (12.0)	30 (12.6)
Liver	31 (13.1)	11 (8.3)	14 (5.9)
Renal	6 (2.5)	6 (4.5)	11 (4.6)
Baseline eGFR < 60 mL/min/1.73 m^2^			
*N*	110	58	108
Cardiac, QT, and arrhythmias	10 (9.1)	6 (10.3)	10 (9.3)
Cardiac, ischemias	10 (9.1)	8 (13.8)	23 (21.3)
Liver	18 (16.4)	4 (6.9)	10 (9.3)
Renal	16 (14.5)	5 (8.6)	14 (13.0)
All patients			
*N*	348	191	347
Cardiac, QT, and arrhythmias	38 (10.9)	20 (10.5)	35 (10.1)
Cardiac, ischemias	37 (10.6)	24 (12.6)	53 (15.3)
Liver	49 (14.1)	15 (7.9)	24 (6.9)
Renal	22 (6.3)	11 (5.8)	25 (7.2)

A patient with multiple occurrence of an AE under a preferred term is counted only once under the preferred term. MedDRA version 18. 0 has been used for the reporting of adverse events. Narrow SMQ search: liver (hepatic failure, fibrosis, and cirrhosis and other liver damage related conditions; hepatitis, non-infectious; liver-related investigations, signs and symptoms; cholestasis and jaundice of hepatic origin). Broad SMQ search: cardiac, QT and arrhythmias (arrhythmia related investigations,signs and symptoms, cardiac arrhythmia terms [including bradyarrhythmias and tachyarrhythmias]); cardiac, ischemias (ischemic heart disease); renal (acute renal failure)

#### Cardiac safety

Adverse events related to cardiac arrhythmias or QT prolongations were comparable across treatment groups (Table 4). For the ECG findings at EOS, mean heart rate declined over the treatment period; however, PR interval increased and a marginal increase was also observed in the duration of QRS complex (heart rate: OCT20 = −3.4 bpm; OCT30 = −4.4 bpm; placebo = −3.3 bpm; PR interval: OCT20 = 6.1 ms; OCT30 = 6.9 ms; placebo = 9.0 ms; QRS duration: OCT20 = 3.0 ms; OCT30 = 2.9 ms; placebo = 1.7 ms). The changes in Fridericia’s corrected QT interval (QTcF) over time were similar across treatment groups when compared to the placebo arm: OCT20 = 7.6 ms; OCT30 = 7.1 ms; placebo = 4.2 ms. At the EOS assessment, the QTcF outliers did not exceed 480 ms or 500 ms in any of the treatment groups. The incidences of categorical changes of QTcF were as follows: +30 ms: OCT20 = 13.3; OCT30 = 8.1; placebo = 11.6, and for +60 ms: OCT 20 = 1.7; OCT30 = 1.5; placebo = 0.6.

Events potentially related to Torsades de pointes (fall, syncope, cardiac arrest, and loss of consciousness) were similar between the OCT30 and placebo treatment groups. In total, three instances of sudden death occurred during the trial period: OCT20 = no cases; OCT30 = 2 cases; placebo = 1 case. All three patients (aged 51, 52, and 63 years) had pre-existing hypertension in addition to diabetes and died at home while sleeping. No autopsy reports were available. The investigators assessed the deaths as not related to study treatment.

The overall incidence of AEs related to cardiac ischemia (SMQ, Table 4) was lower in the octreotide treatment arms compared with the placebo arm (OCT20 = 12.6% [24/191]; OCT30 = 10.6% [37/348]; placebo = 15.3% [53/347]). The incidence of events related to coronary artery disease (CAD) and angina pectoris (AP) were also lower in the treatment arms compared with the placebo arm (CAD: OCT20 = 1.6% [3/191], OCT30 = 3.4% [12/348], placebo = 5.8% [20/347]; AP: OCT20 = 2.6% [5/191], OCT30 = 1.4% [5/348], placebo = 4.3% [15/347]). The incidence of events related to myocardial infarction was however, similar between OCT30 and placebo groups and comparatively higher in the OCT20 group (OCT20 = 5.2% [10/191]; OCT30 = 2.9% [10/348]; placebo = 2.9% [10/347]).

Based on the individual preferred terms, the incidences of left ventricular hypertrophy (OCT20 = 1.6% [3/191]; OCT30 = 1.1% [4/348]; placebo = 3.2% [11/347]) and increased blood pressure (OCT20 = 2.6% [5/191]; OCT30 = 1.7% [6/348]; placebo = 4.9% [17/347]) were lower with octreotide than for placebo.

#### Renal safety

The mean baseline eGFR was evenly distributed across treatment groups (OCT 20, OCT30 and placebo). There was a gradual decline in eGFR over the treatment period and the trend was similar across all the 3 treatment groups. The overall incidences of renal AEs (identified by SMQ search) were comparable between treatment groups (OCT20 = 5.8% [11/191]; OCT30 = 6.3% [22/348]; placebo = 7.2% [25/347]). The most commonly reported event term identified by renal SMQ search was renal failure (OCT20 = 2.6% [5/191], OCT30 = 3.4% [12/348]; placebo = 3.7% [13/347]).

#### Hepatic safety

The incidence of AEs identified using the four liver SMQs was higher in the OCT30 group than placebo (OCT30 vs. placebo = 0.14 vs. 0.07; RR = 2.04 [95% CI, 1.28–3.26]). Comparable incidences in liver-related AEs were observed in the OCT20 and placebo groups (OCT20 vs. placebo = 0.08 vs. 0.07; RR = 1.50 [95% CI, 0.69–3.25]).

The most common event was hepatic steatosis, occurring in 5.2% (10/191) patients and 8.0% (28/348) patients in the OCT20 and OCT30 groups, respectively, vs. 4.9% (17/347) patients receiving placebo. The only other AE occurring in >1% of the patients in any treatment group was hepatomegaly (OCT20 = 0; OCT30 = 1.7% [6/348]; placebo = 0.6% [2/347]).

##### Deaths/serious adverse events

The incidences of death for any cause during the study ranged from 4 to 6.3% between groups (OCT20 = 6.3% [12/191]; OCT30 = 4.0% [14/348]; placebo = 4.6% [16/347]). In the events with a reported fatal outcome, cardiac disorder was identified as the most common cause with incidences of 4.2% (8/191) on OCT20, 2% (7/348) on OCT30 and 2% (7/347) on placebo, respectively.

Overall, the frequency of serious adverse events (SAEs) was similar between treatment groups, but the SAEs suspected to be related to study drug were reported more frequently in the active treatment groups (OCT20 = 7.9% [15/191]; OCT30 = 10.1% [35/348]; placebo = 3.5% [12/347]). The most common SAEs suspected to be drug-related (>0.5% of all study patients) were cholelithiasis (OCT20 = 2.6% [5 of 191], OCT30 = 4.3% [15 of 348], placebo = 0.3% [1/347]); hypoglycemia (OCT20 = 2.1% [4/191], OCT30 = 1.7% [6/348], placebo = 0%); cholecystitis (OCT20 = 0.5% [1/191], OCT30 = 1.1% [4/348], placebo = 0.6% [2/347]), diarrhea (OCT20 = 1.0% [2/191], OCT30 = 0.6% [2/348], placebo = 0.6% [2/347]), abdominal pain (OCT20 = 0.5% [1/191], OCT30 = 0.9% [3/348], placebo = 0.3% [1/347]), bile duct stone (OCT20 = 0.5% [1/191], OCT30 = 0.6% [2/348], placebo = 0.6% [2/347]), and cholecystitis chronic (OCT20 = 1.0% [2/191], OCT30 = 0.9% [3/348], placebo = 0%). Only one case of cardiac SAE was suspected to be study drug-related (OCT30 group = 0.3% [1/348]) and the respective incidences of cardiac SAEs that were not suspected to be study drug related were as follows: OCT20 = 9.4% (18/191); OCT30 = 9.2% (32/348); placebo = 14.7% (51/347). Of note, the renal and urinary disorders related SAEs were not suspected to be study drug-related (OCT20 = 7.9% [15/191]; OCT30 = 3.4% [12/348]; placebo = 4.3% [15/347]).

## Discussion

The safety profile of octreotide is well established [6, 16–21]. However the long-term effects of octreotide on hepatic, cardiac, and renal safety have only been assessed in uncontrolled settings until now. The pooled analysis of data from the long-term safety follow-up of the two clinical trials presented in the current study, allows for a unique safety review of octreotide. The safety data were both placebo-controlled and had a long median duration of treatment of over 3.5 years. Furthermore, the underlying study population of patients with diabetic retinopathy (not an approved indication for octreotide) provided an opportunity to assess the long-term safety of octreotide in a high-risk population which would potentially be at increased risk for adverse outcomes.

Based on the RR analysis, the long-term use of octreotide was not associated with an increased cardiac risk in these patients regardless of the presence of other risk factors. The incidences of cardiac events (QT prolongation and arrhythmia) for the OCT20 and OCT30 groups were comparable to placebo (OCT20, RR = 1.11 [95% CI, 0.61–2.03]; OCT30, RR = 1.09 [95% CI, 0.70–1.68]). However, the incidences of cardiac ischemic events were lower in the octreotide treated patients and the risk of these events decreased upon octreotide treatment compared with placebo (OCT20, RR = 0.80 [95% CI, 0.49–1.32]; OCT30, RR = 0.69 [95% CI, 0.47–1.03]). It is noteworthy that fewer coronary artery disease events occurred on treatment with octreotide than placebo. No SAEs reported in the SOC of cardiac disorders were suspected to be related to octreotide.

The frequency of liver-related AEs was higher in patients receiving octreotide (OCT20 and OCT30) than in the placebo group. As one might expect, the overall prevalence of hepatic steatosis in this study was high, as this condition is associated with diabetes [22–24]. In the general population, the prevalence of fatty liver is estimated to be 20%, while it could be as high as 70% in obese patients with type 2 diabetes mellitus [22]. This event was the most frequent in all groups including the placebo group and appeared to occur more frequently in the octreotide treatment groups, although this observation was noted in only one of the studies (study 1). The incidence of other liver-related events was low and showed no particular clinical concern for severity. Only a few events of transaminase increase were reported as AEs. Liver chemistry elevations were generally mild and transient, and occurred within the first months of therapy. In the majority of these cases, the liver laboratory values returned to normal without discontinuation of octreotide. Transient and slight elevations of liver enzymes are a well-known class effect observed with SSAs. Other hepatic events (observed in patients on octreotide treatment [OCT20 and OCT30]: hepatomegaly, *n* = 6; cholestasis, *n* = 3; hepatic cirrhosis, *n* = 2; ascites, *n* = 2; portal hypertensive gastropathy, *n* = 1; hepatic encephalopathy, *n* = 1; hepatitis, *n* = 1; non-alcoholic steatohepatitis, *n* = 1) occurred at very low numbers, and no clinically relevant imbalance was seen between octreotide and placebo groups.

The overall renal function profile was similar for both studies. The RR of renal events in both the octreotide treatment groups was not significant compared with the placebo treatment group. This was also apparent from the post-baseline change in eGFR values; indicating that the effect of disease on the renal function surpassed any treatment effect. This is consistent with data from the literature indicating that octreotide has no detrimental effect on renal hemodynamics and tubular function [25–27].

In summary, the results from these unique long-term placebo-controlled studies confirm the established safety profile of long-acting octreotide in patients with diabetic retinopathy, and also in particular demonstrate the low risk of hepatic, cardiac, and renal toxicity.
